# The Effects of Wearing a Medical Mask on the Masticatory and Neck Muscle Activity in Healthy Young Women

**DOI:** 10.3390/jcm11020303

**Published:** 2022-01-07

**Authors:** Michał Ginszt, Grzegorz Zieliński, Jacek Szkutnik, Marcin Wójcicki, Michał Baszczowski, Monika Litko-Rola, Ingrid Rózyło-Kalinowska, Piotr Majcher

**Affiliations:** 1 Department of Rehabilitation and Physiotherapy, Medical University of Lublin, 20-093 Lublin, Poland; zaklad.rehabilitacji@umlub.pl; 2 Department of Sports Medicine, Medical University of Lublin, 20-093 Lublin, Poland; grzegorz.zielinski@umlub.pl (G.Z.); michal.baszczowski@umlub.pl (M.B.); 3 Independent Unit of Functional Masticatory Disorder, Medical University of Lublin, 20-093 Lublin, Poland; zakladzaburzen@umlub.pl (J.S.); marcin.wojcicki@umlub.pl (M.W.); monika.litko@umlub.pl (M.L.-R.); 4 Department of Dental and Maxillofacial Radiodiagnostics, Medical University of Lublin, 20-093 Lublin, Poland; rozylo.kalinowska@umlub.pl

**Keywords:** COVID-19, SARS-CoV-2, surface electromyography, masticatory muscles, medical mask

## Abstract

The objective of this study was to analyze the influence of wearing a medical mask on masticatory and neck muscle activity in healthy young women. We recruited 66 healthy women aged from 18 to 30 years (mean 23.6 ± 2.3 years). The temporalis anterior (TA), the superficial part of the masseter muscle (MM), the anterior bellies of the digastric muscle (DA), and the middle part of the sternocleidomastoid muscle (SCM) potentials were recorded at rest and during functional activity using an eight-channel device for surface electromyography—BioEMG III^TM^. There was a statistically significant decrease in mean TA activity during medical mask measurement compared to no mask examination at rest (2.16 µV vs. 2.58 µV; *p* = 0.05; ES = 0.2). Significant decreases in resting RMS values were also observed during the medical mask phase in comparison to no mask examination concerning the left MM (1.75 µV vs. 2.17 µV; *p* = 0.01; ES = 0.3), and mean bioelectrical activity of the MM (1.81 µV vs. 2.15 µV; *p* = 0.02; ES = 0.2). The differences between the two conditions did not reach the assumed significance level (*p* > 0.05) in terms of other indices. Wearing a medical mask has a small effect on decreasing the resting potentials of the temporalis anterior and masseter muscles without changing the parameters of activity and asymmetry within the stomatognathic system.

## 1. Introduction

The severe acute respiratory syndrome coronavirus 2 (SARS-CoV-2) is a single-stranded RNA virus that can be transmitted from human to human through respiratory secretions, causing various clinical symptoms leading to coronavirus disease 2019 (COVID-19) [1]. It is known that COVID-19 can be transmissible from presymptomatic, paucisymptomatic, and asymptomatic people. Thus, reducing disease spread requires preventive management of COVID-19, which includes vaccination, quarantine, personal protective equipment (e.g., face masks, gloves), hand hygiene, and physical distancing [2,3]. The preponderance of scientific evidence suggests that face mask wearing lowers transmissibility per contact by reducing transmission of infected respiratory particles [4]. Moreover, the face mask may reduce the inoculum of the virus to which a mask-wearer is exposed, which will result in milder disease [5,6]. Therefore, face masks are recommended to reduce the chances that the wearer spreads SARS-CoV-2, especially in healthcare settings [7,8]. On the other hand, many countries introduced the requirement to wear face masks in public spaces, making it commonplace in 2021 [9]. 

According to many health and epidemiological benefits of wearing face masks during the COVID-19 pandemic, several studies have examined the possible negative consequences of applying face masks [10]. Scientific reports presented evidenced changes in respiratory physiology of mask wearers with a significant correlation of O_2_ drop [11,12], CO_2_ rise [13,14,15], heart rate increase [16], headache [17,18,19,20,21], and temperature and moisture rise under the face masks [16]. The above-mentioned physiological changes may contribute to headaches during the prolonged mask wearing with a shift towards hypoxia and hypercapnia [10]. On the other hand, several mechanical factors such as the irritation of cervical nerves and associated structures in the neck and head area caused by the face mask straps pressuring the nerve strands also contribute to headaches [18]. As the face mask covers the face and the masticatory muscles, especially the masseter muscle, it is also possible that the activity of these muscle groups will be affected. Moreover, a face mask with a loop around each ear can put pressure on the temporalis muscle. However, the direct influence of the face mask on the activity of the above-mentioned muscles has not yet been scientifically investigated.

Extended wearing of face masks by the general population may lead to relevant effects and consequences in various medical areas. Thus, a comprehensive risk-benefit analysis is critical regarding the potential long-term impacts of face masks. So far, there is a lack of studies analyzing the effect of using the face mask on the muscles within the stomatognathic system. Therefore, in our study of a homogeneous cohort, we tested the effects of wearing medical masks on resting and functional masticatory and neck muscle activity. To the best of our knowledge, this is the first study analyzing changes in electromyographic activity and asymmetry of masticatory and cervical spine muscles during medical mask wearing. We hypothesize that wearing a medical mask significantly influence the activity of the masticatory and neck muscles. 

## 2. Materials and Methods

### 2.1. Study Population

The presented study was carried out between May 2021 and September 2021 at the Department of Functional Masticatory Disorders, Medical University of Lublin, Poland. The measurements were carried out according to the Helsinki Declaration’s recommendations and with the Bioethics Committee’s consent of the Medical University of Lublin (KE-0254/81/2021). All participants were informed about the aim of the study and have given written permission for the research.

We recruited 66 healthy women aged from 18 to 30 years (mean 23.6 ± 2.3 years) basing on following exclusion criteria: the occurrence of headache and cervical spine pain within the month preceding the examination; the occurrence of orofacial pain within the month prior to the test; head and neck injuries within the last six months before the study; previous head and neck surgical treatment within the last six months before the examination; pregnancy; craniofacial trauma; class II and III of the bite according to Angle’s classification; open bite; lack of four support zones in dental arches; lack of more than four teeth within both dental arches; carious or damaged dental tissues; any periodontal pathology; any pathology or asymmetry in craniofacial structures; any form of temporomandibular disorders (TMDs) according to the Research Diagnostic Criteria for Temporomandibular Disorders (RDC/TMD); condition during orthodontic treatment; possession of dental prostheses (regardless of type); Botox therapy; neurological disorders. Moreover, participants unable to wear a medical mask due to an underlying medical condition were not eligible. The clinical RDC/TMD examination was performed by the same experienced dentist specializing in dental prosthetics (author M.L-R.). Next, the ultrasound scanning was performed using M-Turbo ultrasound machine equipped with the 15–16 MHz linear transducer, with scan depth up to 6 cm (SonoSite Inc, Bothell, WA, USA) by experienced dentists specializing in medical radiology (author M.W.). The ultrasound examination was preformed to assess the temporomandibular joint structures and confirm the RDC/TMD examination results.

### 2.2. Study Protocol

**Figure 1:**
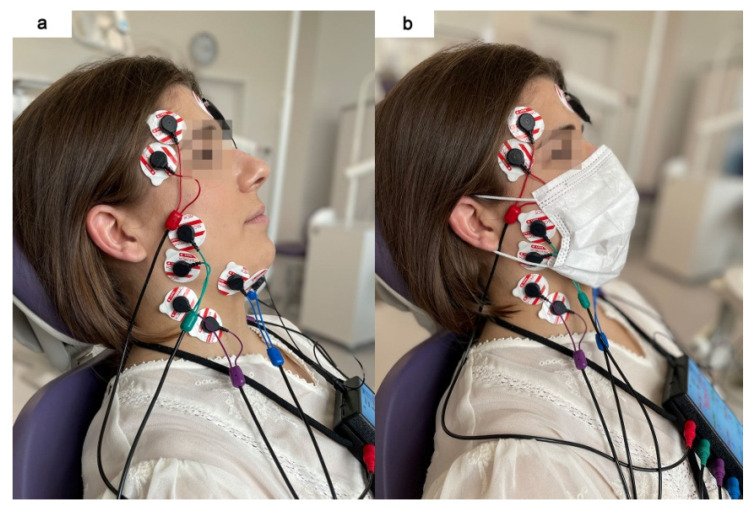
Electromyographic examination during two conditions: without (**a**) and with medical mask (**b**).

The study consisted of two phases, with sEMG measurements of all four masticatory activities of each phase. Participants completed each of the four masticatory activities when they wore no mask and a certified disposable three-layer medical mask (Type II 50PSC, 000-994, Abeba GmbH, St. Ingbert, Germany) with 5 min of rest between measurements. There was a random selection of the initial phase. The medical mask always covered the subject’s nose and mouth, as presented in Figure 1. The position of the mask was the same for all subjects, and it did not cause any discomfort for the participants.

The muscle activity was recorded using an eight-channel device for surface electromyography—BioEMG III^TM^ (BioResearch Associates, Inc., Milwaukee, WI, USA). Electromyographic signals were obtained from eight channels. Masticatory and neck muscle activity was measured during four activities: during resting mandibular position (ten seconds), during clenching in intercuspal position (three times for three seconds each, with two seconds of rest between), during clenching on dental cotton rolls between teeth (three times for three seconds each, with two seconds of rest between) and during active maximum mouth opening (three times for three seconds each, with two seconds of rest between). The average of the three measurements of each variable was used for analysis.

### 2.3. Electromyographic Examination

The sEMG examinations were conducted between 8 and 12 a.m. to minimize the influence of daily fluctuations of muscle activity. The electromyographic measurements were carried out in the same dental chair in a sitting position (the body perpendicular to the ground, the head resting on the chair’s headrest, and the lower limbs upright and arranged parallel). The height of the headrest was adjusted individually to set the head, neck, and torso of the subjects in a straight line.

Before placing the surface electrodes, the skin was cleaned with 90% ethanol solution to reduce skin impedance. Next, surface electrodes (Ag/AgCl with a diameter of 30 mm and a conductive surface of 16 mm (SORIMEX, Toruń, Poland) were placed bilaterally following the course of the muscle fibers of the temporalis anterior (TA), the superficial part of the masseter muscle (MM), the anterior bellies of the digastric muscle (DA), and the middle part of the sternocleidomastoid muscle (SCM) according to the SENIAM (Surface EMG for Non-Invasive Assessment of Muscles) guidelines [22]. Placing surface electrodes was performed by the same physiotherapist (author G.Z.). The reference electrode was placed on the forehead, in the center of the frontal bone. The arrangement of the electrodes symmetrically on the skin covering the examined muscles on both sides was preceded by palpation of the muscles during mandibular and head/neck movements. The electrodes on the superficial masseter muscle were located along the line from the mandible angle to the inferior border of the zygomatic bone. The electrodes on the anterior part of the temporal muscle were arranged along a perpendicular line from the superior border of the zygomatic bone to a cranial bone (in the projection of the sphenoid bone). The electrodes on the anterior bellies of the digastric muscle were placed approximately 1 cm medial to the base of the mandible. The electrodes on the sternocleidomastoid muscle were placed in the middle part of the muscle belly. The edges of the surface electrodes were in contact to maintain a constant spacing between the electrodes, as presented in Figure 1 [22].

### 2.4. sEMG Signal Processing and Normalization

**Figure 2:**
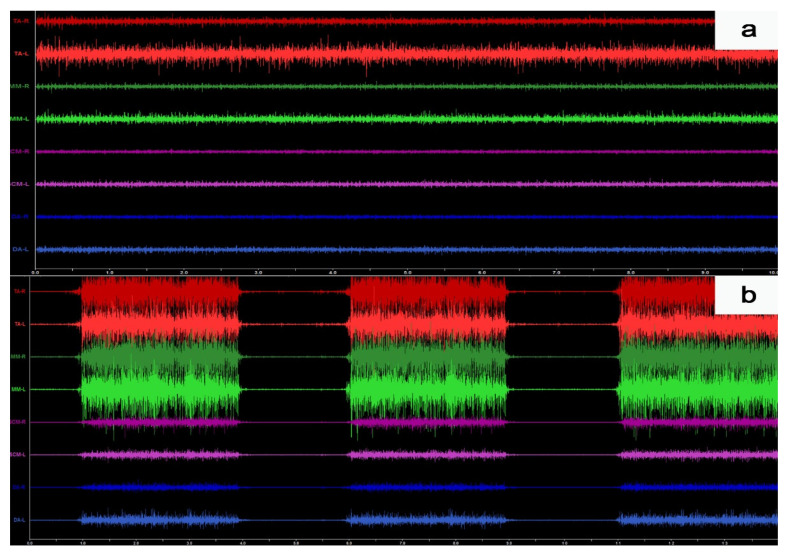
Example of the surface electromyography traces during resting activity (**a**) and maximum voluntary clenching in the intercuspal position (**b**).

Microvolt signals were amplified with minimal noise to 5000 times their original levels. The noise was reduced by 40 dB using the Noise Buster digital filtering in the BioPAK Measurement System, which automatically removes 99% of any remaining 50/60 Hz noise. The automatic processing of the electromyographic signal based on root mean square (RMS) calculations in the BioPAK program allowed us to obtain the average bioelectric values, which were then used for the sEMG analysis (Figure 2). Moreover, all the electromyographic signals were confirmed visually before each RMS processing.

The following asymmetry (AsI) and activity (AcI) calculations were used for the normalization of the mean bioelectric activity from the average temporalis anterior, masseter, digastric, and sternocleidomastoid muscles RMS potentials, according to Naeije et al. [23] and Ferrairo et al. [24]. The AsI varies between +100 and −100, with an AsI of +100 describing only right muscle activity, −100 meaning only left muscle activity, and 0 meaning equal left and right muscle activity. The AcI varies between +100 and −100. The negative (−) values indicate the predominance of the temporalis anterior and positive (+) values suggest a masseter muscle advantage [25].

Temporalis anterior asymmetry index (AsI_TA_) = (TA_right_ − TA_left_) / (TA_right_ + TA_left_) × 100 (1)

Masseter muscle asymmetry index (AsI_MM_) = (MM_right_ − MM_left_) / (MM_right_ + MM_left_) × 100 (2)

Digastric muscle asymmetry index (AsI_DA_) = (DA_right_ − DA_left_) / (DA_right_ + DA_left_) × 100 (3)

Sternocleidomastoid muscle asymmetry index (AsI_SCM_) = (SCM_right_ − SCM_left_) / (SCM_right_ + SCM_left_) × 100 (4)

Activity index right-sided (AcI_R_) = (MM_right_ − TA_right_) / (MM_right_ + TA_right_) × 100 (5)

Activity index left-sided (AcI_L_) = (MM_left_ − TA_left_) / (MM_left_ + TA_left_) × 100 (6)

Activity index both-sided (AcI_Total_) = (MM_right_ + MM_left_ − TA_right_ − TA_left_) / (MM_right_ + MM_left_ + TA_right_ + TA_left_) × 100 (7)

### 2.5. Statistical Analysis

The checklist developed by the Strengthening the Reporting of Observational Studies in Epidemiology (STROBE) initiative was used to assess the methodological quality of the presented study [26]. The repeatability of the sEMG protocol was proved by duplicate sEMG measurements on 10 participants. The two independent sEMG measurements were separated by 5 min rest between all masticatory activities. There were no significant differences (*p* > 0.05) between repeated sEMG records in all analyzed variables (resting mandibular position, maximum voluntary clenching, maximum voluntary clenching on cotton rolls between teeth, maximum mouth opening).

Statistical analysis was carried out using Statistica 13.3 analytics software (TIBCO Software Inc., Palo Alto, CA, USA). First, the normality of the distribution of variables was verified using the Shapiro–Wilk test and the Kolmogorov–Smirnov test (with Lillierfors correction). The Student *t*-test (T) or Mann–Whitney U test (Z) was used depending on the distribution. The significance level was set at 0.05. Effect sizes were determined for Z-test using the Cohen d method and interpreted as small (0.2), medium (0.5), and large (0.8) effect sizes [27,28].

## 3. Results

### 3.1. RMS sEMG Activity

There was a statistically significant decrease in mean temporalis anterior activity during medical mask measurement compared to no mask examination at rest (2.16 µV vs. 2.58 µV; *p* = 0.05; ES = 0.2). Significant decreases in resting RMS values were also observed during the medical mask phase in comparison to no mask examination concerning the left masseter muscle (1.75 µV vs. 2.17 µV; *p* = 0.01; ES = 0.3), and mean bioelectrical activity of the masseter muscles (1.81 µV vs. 2.15 µV; *p* = 0.02; ES = 0.2). In terms of other indices, the differences between the two conditions did not reach the assumed significance level (*p* > 0.05) (Table 1).

### 3.2. Asymmetry and Activity Indices

Statistical analysis showed that there were no significant differences (*p* > 0.05) between no mask and medical mask measurements in terms of all asymmetry and activity indices during resting and functional masticatory activities (Table 2 and Table 3).

## 4. Discussion

By the end of June 2020, nearly 90% of the global population lived in countries that had laws requiring mask use in public locations, and community mask use was recommended by almost all major public health organizations [4]. Many medical data support the legitimacy of using face masks in public places. The epidemiological model suggests that face masks wearing is most effective at reducing the spread of the virus when compliance is high [29]. Moreover, medical mask wearing lowers transmissibility per contact by reducing transmission of infected respiratory particles [4]. On the other hand, prolonged face mask use may lead to relevant effects and consequences in respiratory physiology [10]. Thus, a comprehensive risk-benefit analysis is critical regarding the potential long-term impacts of face masks.

As the face mask covers the face and the masticatory muscles, especially the masseter muscle, it is also possible that the masseter muscle activity will be changed while wearing the mask. In addition, a mask with a loop around each ear can put pressure on the temporalis muscle. So far, there is a lack of studies analyzing the impact of using the face mask on the muscles within the stomatognathic system. Therefore, we tested the effects of wearing medical masks on resting and functional masticatory and neck muscle activity. Our hypothesis that wearing a medical mask significantly influences the activity of the masticatory and neck muscles seems to be confirmed in the presented research. The obtained results indicate that wearing medical masks is related to changes in masticatory muscle activity during resting mandibular position. Surprisingly, wearing a medical mask while electromyographic measurement yielded a significant decrease in resting temporalis anterior and masseter muscle activity compared to the no mask procedure. However, we cannot clearly explain the significant differences observed between the two conditions within the resting masticatory activity. Changes in the electromyographic patterns of masticatory muscles may be associated with the irritation of cervical nerves and associated structures in the neck and head area caused by the face mask straps and mask loops around the ears [18]. Pietropaoli et al. showed a moderate correlation between electric values and palpation-induced pain of both temporalis anterior and masseter muscles [30]. Previous studies indicated the associations between myofascial pain and increased masticatory muscle activity during rest [31,32], which is clearly in opposition to our findings. Moreover, the position of the mask did not cause any discomfort for the participants in our study. Hence the hypothesis that mask-induced discomfort affects muscle activity does not fit the model of pain-induced muscle activity. On the other hand, there were no significant differences in the asymmetry and activity indices between no mask and medical mask measurements. More specifically, changes within temporalis anterior and masseter muscle bioelectric activity did not affect the electromyographic balance among masticatory muscle activity at rest. Reorganization of muscle activity within masticatory muscles may occur in the case of a pain response or abnormal electromyographic activity in chronic TMDs [30,33]. In our study, the temporalis anterior and masseter muscles have similar properties of activity and symmetry regardless of the mask. However, in our opinion, the changes in RMS electromyographic parameters while wearing the medical mask deserve attention and further research to define and validate this mechanism.

As a final comment, we emphasize that there were only significant differences within RMS muscle activity between no mask and medical mask measurements, without changes within the activity and asymmetry parameters. Therefore, we suggest further studies investigating the long-term effect of wearing a medical mask on the activity of the masticatory muscles.

Our study has several limitations that could be addressed in future work. Firstly, a generalization of our findings is limited by the short-term follow-up used in the presented research. Therefore, a more extended observation period is recommended to determine the long-term effects of wearing medical masks. Secondly, the study sample consists of a homogeneous group. We decided to include only healthy young women in the presented research to minimize the influence of gender, age, and health factors on the study results. Thus, future studies should include the male population with an expanded age range. Moreover, it would be worth assessing whether the medical mask influences the symptoms of TMDs and episodes of bruxism in patients with masticatory dysfunctions. Thirdly, the diagnostics criteria for TMDs were replaced by DC/TMDs in 2014. However, in our study, the previous version was used. There is no validated Polish version of the DC/TMDs so far. Therefore, the RDC/TMDs protocol was used.

## 5. Conclusions

Wearing a face mask has a small effect on decreasing the resting potentials of the temporalis anterior and masseter muscles without changing the parameters of activity and asymmetry within the stomatognathic system.

## Figures and Tables

**Table 1:** The comparison of the root mean square (RMS) sEMG activity between no mask and medical mask measurements.

Masticatory Activity	RMS sEMG Activity	No Mask Measurement *n* = 66		Medical Mask Measurement *n* = 66		Test	Test Result	*p*
		M (µV)	SD (µV)	M (µV)	SD (µV)			
Resting activity	TA_R_	2.45	1.71	2.04	1.28	Z	1.84	0.07
	TA_L_	2.71	1.65	2.29	1.48	Z	1.73	0.08
	TA_Mean_	2.58	1.48	2.16	1.20	Z	2.00	0.05 * ES = 0.2
	MM_R_	2.14	1.16	1.87	1.12	Z	1.77	0.08
	MM_L_	2.17	1.06	1.75	0.96	Z	2.75	0.01 * ES = 0.3
	MM_Mean_	2.15	1,.03	1.81	0.96	Z	2.35	0.02 * ES = 0.2
	DA_R_	1.87	0.83	1.89	1.07	Z	0.67	0.50
	DA_L_	1.75	0.76	1.78	0.98	Z	0.30	0.77
	DA_Mean_	1.81	0.77	1.83	0.99	Z	0.53	0.59
	SCM_R_	1.23	0.42	1.13	0.31	Z	1.36	0.17
	SCM_L_	1.34	0.46	1.24	0.41	Z	1.20	0.23
	SCM_Mean_	1.28	0.39	1.18	0.30	Z	1.27	0.20
Maximum voluntary clenching in intercuspal position	TA_R_	136.46	80.88	121.40	72.88	Z	1.12	0.26
	TA_L_	134.46	67.33	121.91	68.21	T	1.06	0.29
	TA_Mean_	135.46	70.05	121.66	68.37	Z	1.12	0.26
	MM_R_	143.25	86.80	120.77	83.17	Z	1.65	0.10
	MM_L_	139.44	85.97	120.28	79.74	Z	1.29	0.20
	MM_Mean_	141.35	83.30	120.53	78.99	Z	1.47	0.14
	DA_R_	22.13	14.76	19.38	13.49	Z	1.12	0.26
	DA_L_	23.50	19.94	18.99	15.29	Z	1.47	0.14
	DA_Mean_	22.82	15.61	19.18	13.47	Z	1.52	0.13
	SCM_R_	10.58	7.57	8.60	6.04	Z	1.60	0.11
	SCM_L_	10.18	8.14	8.52	6.39	Z	1.19	0.23
	SCM_Mean_	10.38	7.51	8.56	5.77	Z	1.40	0.16
Maximum voluntary clenching with dental cotton rolls between teeth	TA_R_	124.13	68.93	125.27	68.69	Z	−0.28	0.78
	TA_L_	122.34	60.90	123.45	64.05	Z	−0.02	0.98
	TA_Mean_	123.24	62.23	124.36	64.27	Z	−0.08	0.93
	MM_R_	160.33	79.99	154.80	75.60	Z	0.35	0.73
	MM_L_	159.39	82.41	151.83	76.70	Z	0.38	0.70
	MM_Mean_	159.86	77.94	153.31	71.58	Z	0.44	0.66
	DA_R_	23.07	11.62	22.00	10.59	Z	0.52	0.61
	DA_L_	23.77	14.10	21.54	13.80	Z	1.37	0.17
	DA_Mean_	23.42	11.80	21.77	11.23	Z	0.98	0.33
	SCM_R_	12.62	7.27	13.30	14.42	Z	0.92	0.36
	SCM_L_	11.73	6.98	11.45	8.27	Z	0.73	0.46
	SCM_Mean_	12.17	6.77	12.38	9.50	Z	0.71	0.48
Maximum active mouth opening	TA_R_	7.00	3.70	9.49	19.02	Z	−0.27	0.79
	TA_L_	6.77	3.96	13.00	48.71	Z	0.03	0.98
	TA_Mean_	6.89	3.42	11.25	25.97	Z	−0.39	0.70
	MM_R_	9.07	8.57	10.71	11.52	Z	−0.46	0.64
	MM_L_	8.29	5.82	9.45	7.19	Z	−0.29	0.77
	MM_Mean_	8.68	6.93	10.08	8.93	Z	−0.45	0.65
	DA_R_	74.54	36.91	80.72	39.30	Z	−0.93	0.35
	DA_L_	75.98	38.99	83.95	40.66	Z	−1.21	0.23
	DA_Mean_	75.26	36.10	82.33	37.67	Z	−1.04	0.30
	SCM_R_	8.90	6.53	10.67	10.92	Z	−0.47	0.64
	SCM_L_	8.72	7.29	10.36	10.80	Z	−0.73	0.47
	SCM_Mean_	8.81	6.63	10.52	10.44	Z	−0.57	0.57

TA—temporalis anterior; MM—masseter muscle; DA—digastric muscle; SCM—sternocleidomastoid muscle; R—right side; L—left side; M—mean; SD—standard deviation; ES—effect size; * Significant difference.

**Table 2:** The comparison of the mean asymmetry index (AsI) between no mask and medical mask measurements.

Masticatory Activity	Asymmetry Index	No Mask Measurement *n* = 66		Medical Mask Measurement *n* = 66		Z	*p*
		M	SD	M	SD		
Resting activity	AsI_TA_	−4.59	25.32	−3.96	24.09	0.06	0.95
	AsI_MM_	−1.12	16.96	2.33	18.66	−0.91	0.36
	AsI_DA_	2.50	10.06	2.00	11.42	0.42	0.68
	AsI_SCM_	−4.19	14.05	−3.96	13.67	−0.08	0.93
Maximum voluntary clenching in intercuspal position	AsI_TA_	−0.43	19.97	0.19	19.47	−0.25	0.80
	AsI_MM_	2.92	17.51	1.51	19.13	0.47	0.64
	AsI_DA_	0.07	22.82	2.17	20.24	−0.48	0.63
	AsI_SCM_	3.08	18.49	0.93	20.33	0.50	0.62
Maximum voluntary clenching with dental cotton rolls between teeth	AsI_TA_	−0.57	14.67	0.55	14.43	−0.50	0.62
	AsI_MM_	0.68	14.36	1.60	15.60	−0.28	0.78
	AsI_DA_	−0.69	18.04	2.85	17.45	−1.31	0.19
	AsI_SCM_	4.15	17.72	3.80	20.90	0.36	0.72
Maximum active mouth opening	AsI_TA_	2.06	18.39	2.73	25.14	0.17	0.86
	AsI_MM_	0.64	19.31	2.23	16.54	−0.33	0.74
	AsI_DA_	−0.62	11.96	−1.51	13.59	0.36	0.72
	AsI_SCM_	1.93	16.59	0.98	17.79	0.52	0.61

AsI_TA_—Asymmetry index for temporalis anterior; AsI_MM_—Asymmetry index for masseter muscle; AsI_DA_—Asymmetry index for digastric muscle; AsI_SCM_—Asymmetry index for sternocleidomastoid muscle.

**Table 3:** The comparison of the mean activity index (AcI) between no mask and medical mask measurements.

Masticatory Activity	Activity Index	No Mask Measurement *n* = 66		Medical Mask Measurement *n* = 66		Test	Test Result	*p*
		M	SD	M	SD			
Resting activity	AcI_R_	−4.05	30.86	2.79	28.50	T	−0.24	0.81
	AcI_L_	−7.71	31.63	8.42	32.30	T	0.13	0.90
	AcI_Total_	−6.94	29.36	−6.34	29.31	T	−0.12	0.91
Maximum voluntary clenching in intercuspal position	AcI_R_	0.08	25.20	−4.78	25.86	Z	1.19	0.23
	AcI_L_	−3.05	26.29	−6.21	24.45	Z	0.91	0.36
	AcI_Total_	−1.66	22.21	−5.47	21.97	Z	1.04	0.30
Maximum voluntary clenching with dental cotton rolls between teeth	AcI_R_	13.11	21.64	11.87	20.77	T	0.34	0.74
	AcI_L_	12.05	18.18	10.88	18.30	Z	0.37	0.71
	AcI_Total_	12.72	16.99	11.57	16.63	Z	0.35	0.72

AcI_R_—Activity index right-sided; AcI_L_—Activity index left-sided; AcI_Total_—Activity index both-sided.

## Data Availability

The data presented in this study are available on request from the corresponding author.

## References

[1] Chan J.F.-W., Yuan S., Kok K.-H., To K.K.-W., Chu H., Yang J., Xing F., Liu J., Yip C.C.-Y., Poon R.W.-S. (2020). A Familial Cluster of Pneumonia Associated with the 2019 Novel Coronavirus Indicating Person-to-Person Transmission: A Study of a Family Cluster. Lancet.

[2] Rahmani A.M., Mirmahaleh S.Y.H. (2021). Coronavirus Disease (COVID-19) Prevention and Treatment Methods and Effective Parameters: A Systematic Literature Review. Sustain. Cities Soc..

[3] Fowlkes A., Gaglani M., Groover K., Thiese M.S., Tyner H., Ellingson K., Cohorts H.-R. (2021). Effectiveness of COVID-19 Vaccines in Preventing SARS-CoV-2 Infection Among Frontline Workers before and during B.1.617.2 (Delta) Variant Predominance—Eight U.S. Locations, December 2020–August 2021. MMWR Morb. Mortal. Wkly. Rep..

[4] Howard J., Huang A., Li Z., Tufekci Z., Zdimal V., van der Westhuizen H.-M., von Delft A., Price A., Fridman L., Tang L.-H. (2021). An Evidence Review of Face Masks against COVID-19. Proc. Natl. Acad. Sci. USA.

[5] Chan J.F.-W., Yuan S., Zhang A.J., Poon V.K.-M., Chan C.C.-S., Lee A.C.-Y., Fan Z., Li C., Liang R., Cao J. (2020). Surgical Mask Partition Reduces the Risk of Noncontact Transmission in a Golden Syrian Hamster Model for Coronavirus Disease 2019 (COVID-19). Clin. Infect. Dis. Off. Publ. Infect. Dis. Soc. Am..

[6] Gandhi M., Beyrer C., Goosby E. (2020). Masks Do More Than Protect Others During COVID-19: Reducing the Inoculum of SARS-CoV-2 to Protect the Wearer. J. Gen. Intern. Med..

[7] Coclite D., Napoletano A., Gianola S., Del Monaco A., D’Angelo D., Fauci A., Iacorossi L., Latina R., Torre G.L., Mastroianni C.M. (2020). Face Mask Use in the Community for Reducing the Spread of COVID-19: A Systematic Review. Front. Med..

[8] Bartoszko J.J., Farooqi M.A.M., Alhazzani W., Loeb M. (2020). Medical Masks vs N95 Respirators for Preventing COVID-19 in Healthcare Workers: A Systematic Review and Meta-Analysis of Randomized Trials. Influenza Other Respir. Viruses.

[9] Royo-Bordonada M.A., García-López F.J., Cortés F., Zaragoza G.A. (2021). Face Masks in the General Healthy Population. Scientific and Ethical Issues. Gac. Sanit..

[10] Kisielinski K., Giboni P., Prescher A., Klosterhalfen B., Graessel D., Funken S., Kempski O., Hirsch O. (2021). Is a Mask That Covers the Mouth and Nose Free from Undesirable Side Effects in Everyday Use and Free of Potential Hazards?. Int. J. Environ. Res. Public Health.

[11] Pifarré F., Zabala D.D., Grazioli G., i Maura I.D.Y. (2020). COVID-19 and Mask in Sports. Apunt. Sports Med..

[12] Beder A., Büyükkoçak U., Sabuncuoğlu H., Keskil Z.A., Keskil S. (2008). Preliminary Report on Surgical Mask Induced Deoxygenation during Major Surgery. Neurocir. Astur. Spain.

[13] Rebmann T., Carrico R., Wang J. (2013). Physiologic and Other Effects and Compliance with Long-Term Respirator Use among Medical Intensive Care Unit Nurses. Am. J. Infect. Control.

[14] Roberge R.J., Coca A., Williams W.J., Powell J.B., Palmiero A.J. (2010). Physiological Impact of the N95 Filtering Facepiece Respirator on Healthcare Workers. Respir. Care.

[15] Georgi C., Haase-Fielitz A., Meretz D., Gäsert L., Butter C. (2020). The Impact of Commonly-Worn Face Masks on Physiological Parameters and on Discomfort During Standard Work-Related Physical Effort. Dtsch. Arzteblatt Int..

[16] Li Y., Tokura H., Guo Y.P., Wong A.S.W., Wong T., Chung J., Newton E. (2005). Effects of Wearing N95 and Surgical Facemasks on Heart Rate, Thermal Stress and Subjective Sensations. Int. Arch. Occup. Environ. Health.

[17] Rosner E. (2020). Adverse Effects of Prolonged Mask Use among Healthcare Professionals during COVID-19. J. Infect. Epidemiol..

[18] Ong J.J.Y., Bharatendu C., Goh Y., Tang J.Z.Y., Sooi K.W.X., Tan Y.L., Tan B.Y.Q., Teoh H.-L., Ong S.T., Allen D.M. (2020). Headaches Associated With Personal Protective Equipment—A Cross-Sectional Study Among Frontline Healthcare Workers During COVID-19. Headache.

[19] Jacobs J.L., Ohde S., Takahashi O., Tokuda Y., Omata F., Fukui T. (2009). Use of Surgical Face Masks to Reduce the Incidence of the Common Cold among Health Care Workers in Japan: A Randomized Controlled Trial. Am. J. Infect. Control..

[20] Ramirez-Moreno J.M., Ceberino D., Gonzalez Plata A., Rebollo B., Macias Sedas P., Hariramani R., Roa A.M., Constantino A.B. (2021). Mask-Associated “de Novo” Headache in Healthcare Workers during the COVID-19 Pandemic. Occup. Environ. Med..

[21] Bharatendu C., Ong J.J.Y., Goh Y., Tan B.Y.Q., Chan A.C.Y., Tang J.Z.Y., Leow A.S., Chin A., Sooi K.W.X., Tan Y.L. (2020). Powered Air Purifying Respirator (PAPR) Restores the N95 Face Mask Induced Cerebral Hemodynamic Alterations among Healthcare Workers during COVID-19 Outbreak. J. Neurol. Sci..

[22] Hermens H.J., Freriks B., Disselhorst-Klug C., Rau G. (2000). Development of Recommendations for SEMG Sensors and Sensor Placement Procedures. J. Electromyogr. Kinesiol..

[23] Naeije M., McCarroll R.S., Weijs W.A. (1989). Electromyographic Activity of the Human Masticatory Muscles during Submaximal Clenching in the Inter-Cuspal Position. J. Oral Rehabil..

[24] Ferrario V.F., Sforza C., Miani A., D’Addona A., Barbini E. (1993). Electromyographic Activity of Human Masticatory Muscles in Normal Young People. Statistical Evaluation of Reference Values for Clinical Applications. J. Oral Rehabil..

[25] Ginszt M., Zieliński G. (2021). Novel Functional Indices of Masticatory Muscle Activity. J. Clin. Med..

[26] von Elm E., Altman D.G., Egger M., Pocock S.J., Gøtzsche P.C., Vandenbroucke J.P., STROBE Initiative (2008). The Strengthening the Reporting of Observational Studies in Epidemiology (STROBE) Statement: Guidelines for Reporting Observational Studies. J. Clin. Epidemiol..

[27] Fritz C.O., Morris P.E., Richler J.J. (2012). Effect Size Estimates: Current Use, Calculations, and Interpretation. J. Exp. Psychol. Gen..

[28] Lakens D. (2013). Calculating and Reporting Effect Sizes to Facilitate Cumulative Science: A Practical Primer for *t*-Tests and ANOVAs. Front. Psychol..

[29] Tian L., Li X., Qi F., Tang Q.-Y., Tang V., Liu J., Li Z., Cheng X., Li X., Shi Y. (2020). Calibrated Intervention and Containment of the COVID-19 Pandemic. arXiv.

[30] Pietropaoli D., Ortu E., Giannoni M., Cattaneo R., Mummolo A., Monaco A. (2019). Alterations in Surface Electromyography Are Associated with Subjective Masticatory Muscle Pain. Pain Res. Manag..

[31] Manfredini D., Cocilovo F., Favero L., Ferronato G., Tonello S., Guarda-Nardini L. (2011). Surface Electromyography of Jaw Muscles and Kinesiographic Recordings: Diagnostic Accuracy for Myofascial Pain. J. Oral Rehabil..

[32] Zieliński G., Byś A., Szkutnik J., Majcher P., Ginszt M. (2021). Electromyographic Patterns of Masticatory Muscles in Relation to Active Myofascial Trigger Points of the Upper Trapezius and Temporomandibular Disorders. Diagnostics.

[33] Mapelli A., Zanandréa Machado B.C., Giglio L.D., Sforza C., De Felício C.M. (2016). Reorganization of Muscle Activity in Patients with Chronic Temporomandibular Disorders. Arch. Oral Biol..

